# Effect of Moisture on the Mechanical Properties of Wood–Plastic Composites Hybridized with Metal Grid Layers

**DOI:** 10.3390/polym15244705

**Published:** 2023-12-14

**Authors:** Srdjan Perišić, Katarina Kalevski, Aleksandar Grujić, Dragutin Nedeljković, Jasna Stajić-Trošić, Vesna Radojević

**Affiliations:** 1 Innovation Center of Faculty of Technology and Metallurgy, University of Belgrade, Karnegijeva 4, 11000 Belgrade, Serbia; sperisic@tmf.bg.ac.rs; 2 Faculty of Stomatology Pancevo, University Business Academy, 21000 Novi Sad, Serbia; katarina.kalevski@sfp.rs; 3 Institute of Chemistry, Technology and Metallurgy, National Institute of the Republic of Serbia, University of Belgrade, Njegoseva 12, 11000 Belgrade, Serbia; dragutin@tmf.bg.ac.rs (D.N.); jtrosic@tmf.bg.ac.rs (J.S.-T.); 4 Faculty of Technology and Metallurgy, University of Belgrade, Karnegijeva 4, 11000 Belgrade, Serbia; vesnar@tmf.bg.ac.rs

**Keywords:** composites, WPC, compression molding, moisture absorption, high impact testing, three-point bending

## Abstract

Wood–plastic composites (WPCs) are some of the most common modern composite materials for interior and exterior design that combine natural waste wood properties and the molding possibility of a thermoplastic polymer binder. The addition of reinforcing elements, binding agents, pigments, and coatings, as well as changes to the microstructure and composition, can all affect the quality of WPCs for particular purposes. To improve the properties, hybrid composite panels of WPCs with 30 wt. % and 40 wt. % of wood content and reinforced with one or three metal grid layers were prepared sequentially by extrusion and hot pressure molding. The results show an average 20% higher moisture absorption for composites with higher wood content. A high impact test (HIT) revealed that the absorbed energy of deformation increased with the number of metal grid layers, regardless of the wood content, around two times for all samples before water immersion and around ten times after water absorption. Also, absorbed energy increases with raised wood content, which is most pronounced in three-metal-grid samples, from 21 J to 26 J (before swelling) and from 15 J to 24 J (after swelling). Flexural tests follow the trends observed by HIT, indicating around 65% higher strength for samples with three metal grid layers vs. samples without a metal grid before water immersion and around 80% higher strength for samples with three metal grid layers vs. samples without a grid after water absorption. The synthesis route, double reinforcing (wood and metal), applied methods of characterization, and optimization according to the obtained results provide a WPC with improved mechanical properties ready for an outdoor purpose.

## 1. Introduction

Wood–plastic composites (WPC) are materials usually composed of up to 80 wt. % wood fibers and particles, often sourced from industrial waste wood and a reduced amount of thermoplastic polymer, predominantly polyethylene (PE) [1]. Hybrid WPCs beside the main components contain additional elements to upgrade the overall properties of the final material. Biodegradable natural fibers mixed into hybrid wood–plastic composites have many benefits, such as low density, high specific strength, good impact and flexural properties, being eco-friendly, and being easy to process at a low cost [2]. Their environmentally friendly and durable nature has led to widespread application in various industries [3] such as the automotive sector [4], civil engineering [3], and interior and exterior design [5,6], successfully replacing inorganic fiber polymer composites. However, a primary drawback is the incompatibility between hydrophilic wood fillers and hydrophobic polymer matrices, predominantly when wood reinforcement content is high [3]. State-of-the-art overviews of the processing, properties, and applications of wood–polymer composites have been conducted and reported by many authors [2,4,7,8,9,10]. Following their achievements and working to improve results so far leads to the balance between a quality product, economic improvement, and the recovery of natural resources. Societal interest in environmental impact detection is growing in lots of branches, including the field of WPCs, which leads to the frequent use of the life cycle assessment (LCA) [11]. WPCs after each life cycle continually produce by-materials with reduced size and qualities (wood, wood particles/fibers, plastics, and composites), which are suitable for reuse in WPCs and, thus, significantly reduce environmental impact [11,12]. 

An increasing amount of polymer waste is globally generated due to the extremely widespread use of polymer materials. Plastic waste can be recycled to produce a new raw material, thereby reducing non-degradable waste; environmental pollution; and, consequently, the loss of natural resources [13,14]. In that sense, the environmental and economic performance of high-density polyethylene and recycled (RPE) polyethylene in specific applications could be compared by LCA [15]. Recycled plastic raw material can be used as new packaging material, sports equipment [16,17], in furniture manufacturing [18], in the automotive industry [19], or as a component of construction materials [20]. 

Waste polyethylene (WPE) removed from the dumptank during production in the petrochemical industry can be recycled or passed on to consumers [7]. Chemical recycling of waste polyethylene is one of the technologies capable of saving 3.5 billion barrels of crude oil globally. The most profitable technology for WPE recycling in 2020 was pyrolysis [21], which produces liquid hydrocarbons with a high yield of 87.5% and is expected to have the largest profit growth up to 2030. From the reducing GHGs and reducing fossil fuel consumption point of view, one of the more acceptable technologies compared with incineration is fast pyrolysis, which produces value-added hydrocarbons [7]. 

Various processing methods could be employed for specific applications of WPC, as reported elsewhere [22,23,24,25,26]. The creep behaviors of WPCs arise from their viscoelastic nature [27] and are influenced by factors such as the composition, compatibility, and nature of the components, as well as moisture. Reduced interfacial adhesion between wood and the polymer matrix results in creep behavior and diminished performance [28]. This incompatibility can be mitigated by using functional polyolefin as a coupling agent, enabling improved stress transfer between the polymer matrix and wood filler [29,30,31]. The properties of a WPC are dependent on the type of polymer, size, geometry, and amount/quantity of reinforcements embedded in the matrix, bonding agents, as well as the optimal processing route [32]. The mechanical properties of WPC can be further enhanced through the addition of reinforcing elements, such as glass fibers [33] or metal wires and grids [34,35,36]. Upgraded mechanical properties of WPC can be achieved by adding alumina particles [37] or using waste recycled PET in a polymer binder [38]. Nevertheless, striking a balance between mechanical performance and environmental sustainability remains a critical concern for hybrid WPC applications [39]. Reinforcing the polymer matrix with particles and fibers impacts material properties (strength, toughness, stiffness, flexibility, durability, etc.) as well as their ecological impact [32,40,41]. The choice of thermoplastic matrices is essential for the thermal stability of hybrid WPC and, subsequently, the thermal degradation of lignocelluloses in wood [39,42]. Polyethylene (PE) is the most commonly used polymer matrix in WPC production routes [43,44,45,46]. Also, other thermoplastic polymers are widely used: polypropylene (PP), polystyrene (PS), poly (vinyl chloride) (PVC), and acrylonitrile butadiene styrene (ABS) [24,28,35]. High-density polyethylene (HDPE) is considered highly crystalline among PEs [36], with a melting temperature of around 130 °C, making it suitable for processing with wood [29]. Intensive exposure to wet environments, predominantly as exterior building elements, leads to special attention to decreasing the level of moisture. Numerous methods for determining water absorption in WPCs are available, including long-term outdoor weathering evaluation [47], exposure to air [48,49], immersion in water [37,50], or placement in weather chambers where environmental conditions can be simulated [51]. Much research in building materials focuses on improving moisture absorption properties and characterizing the impact of coupling agents on the dimensional stability of composites [6,52,53,54,55,56].

Using WPCs for outdoor decking or deck boards is almost always followed by bending because they are set between supports and are often statically loaded by various objects like furniture or are dynamically loaded when walked on. For these types of WPCs, it is important to limit the deflection. In that sense, very important parameters for WPC quality estimation are the maximum (ultimate) strength and modulus of elasticity. Also, impact resistance is an important parameter for WPC application [55]. Fiber-reinforced composite materials do not experience plastic deformations during impact. Elastic deformations may occur near the impact area in the case of low-intensity impacts or material deterioration, such as fiber separation from the matrix, matrix cracking, and fiber breaking. The energy absorbed as a result of impact depends on factors including fiber–matrix bond strength [57,58,59,60]. Laminated structures can be studied using a micro-mechanical approach, which examines failure and damage at the constituent level, or a continuum damage mechanics (CDM) approach, in which the composite material properties have been homogenized and failure and damage can be analyzed at the ply/lamina level [9].

The fracture toughness of composites with polymer matrices is typically assessed through impact puncture tests, which record material responses to impact loads parallel to the thickness direction [61,62,63,64]. This method is frequently employed for out-of-plane fractures, demonstrating material responses to impact loads along the thickness direction. Polymer composites reinforced with particles show that impact energy increases with an increasing quantity of reinforced particles, as discussed previously [65]. 

The motivation of this study is to find a mechanically superior, eco-friendly material with optimal properties for wet, ambient applications. Therefore, an effective technological process is necessary to use natural wood flour as reinforcement for waste polyethylene polymer. As an innovative reinforcement element, layers of metal greed are incorporated into the WPE matrix and make the final WPC product a good base for commercial use. The objective of the research is to study the flexural and impact properties of WPCs after water immersion, examine the effect of absorbed water on structural properties, and use the obtained results to optimize the production parameters.

Compared with individual materials, WPCs can offer more receptive performance, lower manufacturing costs, and pave the way for renewable resource utilization. Due to the fact that design, mechanical properties, and durability are among the most important parameters of quality, WPCs are formulated in a way to meet the needs of consumers by finding the right balance of these properties. The processing of novel WPCs was carried out using two methods: extruding and hot compressing. The correlation between the process parameters, microstructure, and final WPC properties confirms good bonding between the matrix and filler, and the moisture resistance is acceptable for outdoor applications. To this end, a systematic investigation of processing techniques, reinforcement types, and coupling agents is conducted to understand their effects on the mechanical and moisture-absorption properties of hybrid WPCs. Furthermore, advanced characterization methods, such as field emission scanning electron microscopy (FESEM) and Fourier-transform infrared spectroscopy (FTIR), will be employed to analyze the microstructure, chemical structure, and interaction between wood fillers and polymer matrices [66,67,68]. This analysis will provide valuable insights into the factors influencing the mechanical performance and water absorption behavior of these composites.

The findings from this research will contribute to the development of high-performance, environmentally friendly building materials with improved resistance to moisture and enhanced impact properties, ultimately broadening the scope of applications for hybrid WPCs in various industries. 

## 2. Materials and Methods

### 2.1. Materials

The waste polyethylene (WPE) from the dumptank in the reactor (HIP Petrohemija A.D., Pančevo, Serbia) with a processing temperature of 195 °C to 220 °C, a density of 0.936–0.949 g/cm^3^, and a melt flow index of 0.27–0.32 g/10 min was used as a polymer. The used WPE is a copolymer of ethylene and hexene, intended for the production of blown films. The exceptional workability and tenacity of the melt enable the extraction of thin films. Mechanically ground wood, i.e., paper pulp (Metso Paper Oy, Gothenburg, Sweden), was used as wood fiber reinforcement in the WPE matrix. The wood reinforcement was dried in an oven for 24 h at 90 °C to a constant mass in order to eliminate the initial moisture content. The cross-linking bonding agent—Fusabond^®^ W PC-576D, DuPont, Mississauga, ON, Canada, designated as FB—is an anhydride-modified ethylene co-polymer capable of increasing density and flexural modulus of elasticity in WPCs [56,69]. As the metal layer, a steel metal wire grid (Mesh 12, wire diameter of 0.2 mm) was used.

**Figure 1:**
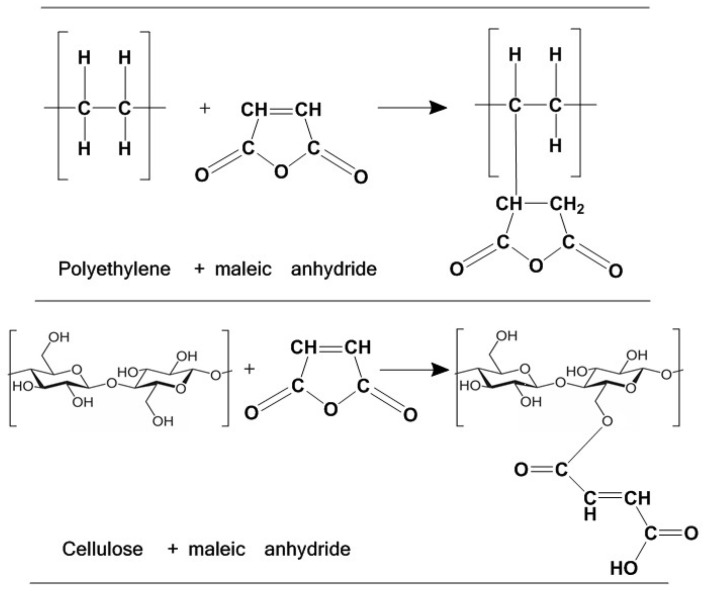
Chemical coupling mechanisms in maleated wood–plastic composites.

The mechanism of the chemical reactions is presented in Figure 1. Polyethylene, one of the most consumed thermoplastic polymers worldwide, is grafted with maleic anhydride [70]. Also, maleic anhydride is joined with cellulose from wood flour [71]. In this way, the used FB compatibilizer affects the creation of strong bonds between the polymer matrix and the filler, which results in excellent physical and mechanical properties of the WPC. Detailed steps of the mechanism of chemical reactions are reported elsewhere [72]. 

### 2.2. Preparation of WPCs

**Figure 2:**
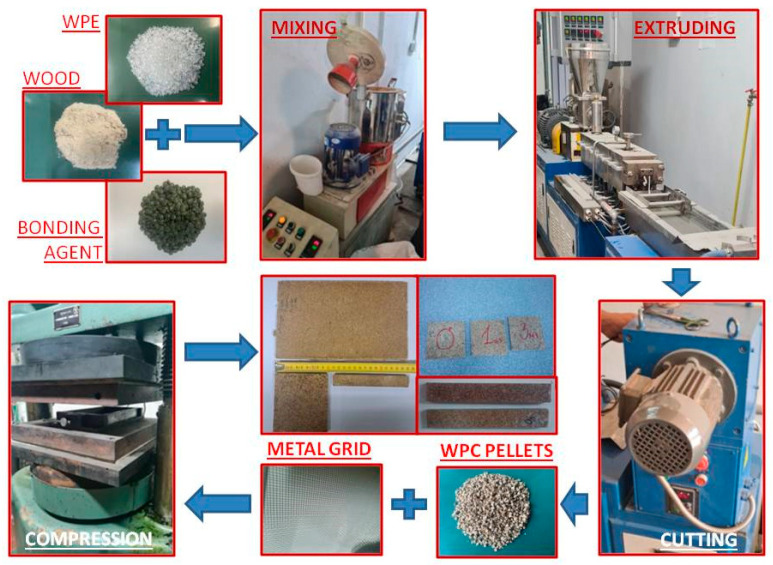
Processing route of WPC materials.

The processing route for WPC is schematically presented in Figure 2. WPE, FB, and wood fibers were mixed in a high-speed mixer at 100 °C for 20 min. Then, the mixture was gravimetrically fed into the co-rotating twin-screw extruder to form long rods with a diameter of 8 mm. A feed was pushed at a screw speed of 80 rpm through six temperature zones between 175 °C and 205 °C. Composite bars were cooled in water and transferred to the universal cutting machine to produce the granules for further hot pressing.

Three series of samples were prepared by hot pressing using a hydraulic press (type “Sojuzzagranpribor”, P-125, Moscow, Russia) with different wood content, with 40 wt. % and 30 wt. % designated as WPC60 and WPC70, respectively (Table 1). The sample series contained different numbers of metal grid layers. The first series is without reinforcement (0), the second is with one metal grid layer (1), and the third is with three metal grid layers (3). The processing of all series was conducted under the same conditions. The predefined content of the WPC was molded using the steel demountable mold (dimensions 140 × 255 mm) at 160 °C under a constant pressure of 2.6 MPa for 20 min (Figure 2). After removing the pressure, the sample was left in the mold for 180 min to cool. For the specimens with metal grid, the amount of WPC pellets was divided into two or more parties and layers were ordered WPC–grid–WPC. The samples were from hybrid composite wafers with dimensions of 100 × 100 mm for HIT measurements and 140 × 20 mm for flexural test, as presented in Figure 2. The thickness of the specimens was 7 mm. Half of the synthesized samples were immersed in water and the other half were subjected to impact and flexural tests.

### 2.3. Methods of Characterization

#### 2.3.1. Chemical Characterization

The Fourier-transform infrared (FT-IR) spectra of the components and WPC sample in the KBr discs were recorded by a BOMEM spectrometer (Hartmann & Braun, MB series, Frankfurt/Main, Germany) with a resolution of 4 cm^−1^ and a transmission mode between 4000 and 400 cm^−1^.

#### 2.3.2. Thermal Analysis

Thermal analysis was conducted on a SDT Q600 (TA Instruments, New Castle, DE, USA) for simultaneous TG/DSC measurements from room temperature to 600 °C at a heating rate of 10 °C/min under a nitrogen flow of 500 mL/min.

#### 2.3.3. Moisture Absorption

An investigation of moisture absorption was performed by immersing samples in a glass filled with deionized water and leaving them in it for 7 days at an ambient temperature. The weight and volume of all samples (WPC60 and WPC70) were measured before and after water immersion.

The water absorption (%) of all the samples was calculated according to the following equation:

(1) $$M_{t}\left(\%\right)=\frac{W_{t}-W_{0}}{W_{0}}\times 100$$

where *M_t_* is the amount of water absorbed at time *t*, and *W_t_* and *W*_0_ are the weight of the sample at time *t* and the initial weight of the sample, respectively.

The swelling of samples is calculated according to the following equation:

(2) $$∆V=\frac{V_{t}-V_{0}}{V_{0}}\times 100$$

where *V_t_* and *V*_0_ are the volume of the sample at time *t* and the initial volume of the sample, respectively.

#### 2.3.4. Impact Test

**Figure 3:**
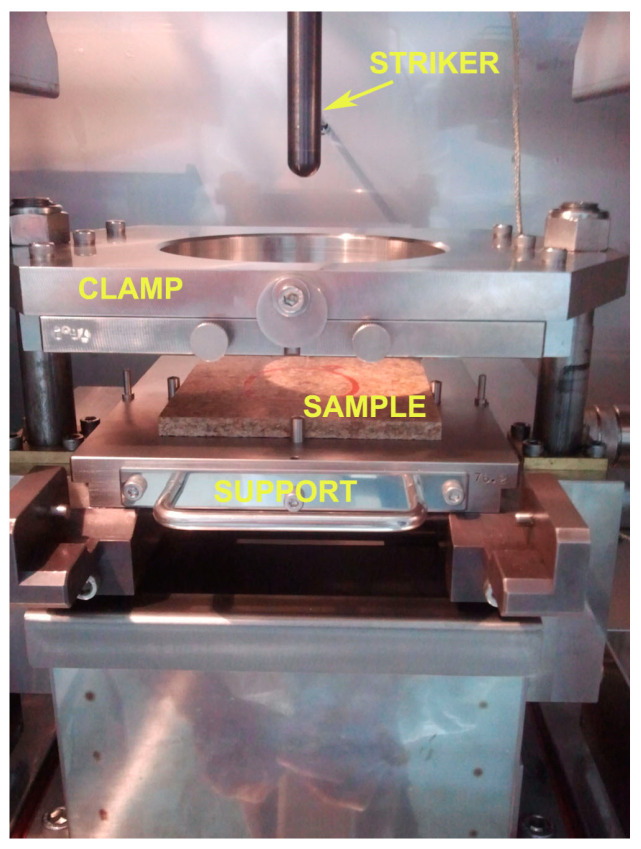
The impact test device setup.

The impact tests were conducted by the high-speed puncture impact (HIT) device (Hydroshot Hits—P10, Shimadzu, Kyoto, Japan), as presented in Figure 3. A clamp with a 40 mm diameter and a pressure of 0.55 MPa was used to fix the sample. The striker head with a diameter of 12.7 mm could be programmed to a specific velocity, height, and depth. The impact velocity was 2 m/s while the force was 10 kN. Using predefined parameters, impact energy could be controlled. The changes in force, velocity, deflection, and energy of impact with time were collected and analyzed.

#### 2.3.5. Flexural Strength Test

The flexural properties were determined using an Instron 8800 universal fast track machine with the crosshead velocity of 0.2 cm/min. A load cell of 1 kN capacity was used for loading measuring. The rectangular-shaped 140 × 20 × 7 mm specimen was cut from a hot-pressed plate to be used in three-point bending tests, as required by ASTM D 790-03 [73]. At least five specimens were tested for each WPC composition, with a distance between the supports of 100 mm. A specimen is loaded by means of a loading nose midway between the supports. 

Performing a flexural test on the homogeneous WPC composite material, where the supports are at distance L while the applied force is at the middle point, the maximum stress can be calculated at any point on the force–deflection curve using the following equation [73]:

(3) $$\sigma_{s}=\frac{3F\cdot L}{2b\cdot d^{2}}$$

where *σ_s_* (MPa) is stress on the outer part at midpoint; *F* (N) is load at a given point on the load–deflection curve; *L* (mm) is distance between supports; *b* (mm) is width of the specimen; and *d* (mm) is thickness of the specimen. 

#### 2.3.6. Surface Characterization

An optical microscope (Leica DM ILM, Wetzlar, Germany) with reflected light and magnification of 50–100 times was used for surface morphology observation of the wood flour and WPC samples. The microscope is equipped with a CCD digital camera connected to a PC for capture and further image processing.

Samples’ fracture surfaces were sputtered with gold to make them more conductive, and they were looked at with a Field Emission Scanning Electron Microscope (FESEM), Tescan Mira 3 XMU, Brno, Czech Republic.

The average diameter of selected fibers and particles was characterized using the ImageProPlus 4.0 Media Cybernetics software package. The mean diameter of grains and fibers is calculated on the basis of 5 FESEM images.

## 3. Results and Discussion

### 3.1. Chemical Properties

**Figure 4:**
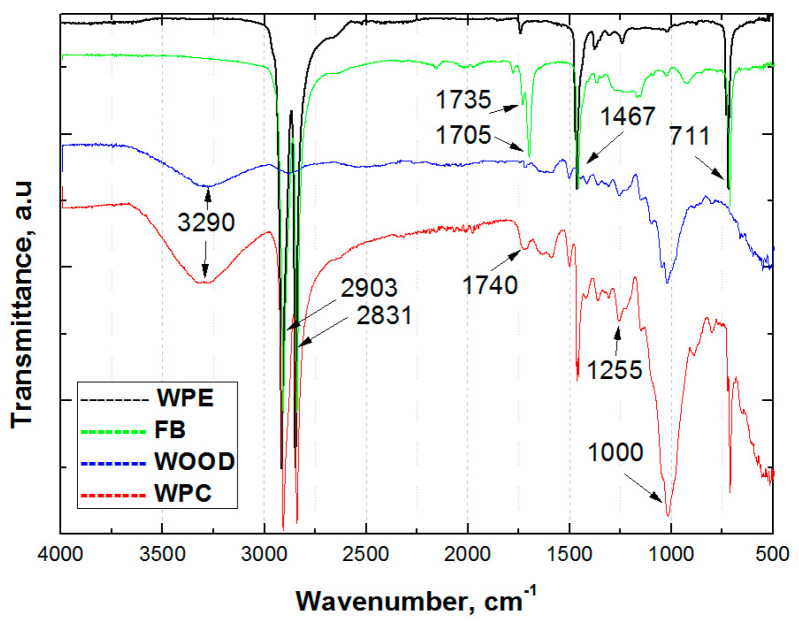
The FT-IR spectra of WPC and components.

The results of FT-IR analysis of the wood flour (wood), bonding agent (FB), polymer matrix (WPE), and wood–plastic composite (WPC) are presented in Figure 4.

The broadband at 3290 cm^−1^ corresponds to O-H stretching vibrations, mainly originating from absorbed water.

The FT-IR spectrum of WPE shows a band associated with aliphatic C-H stretching of the –CH2- functional group at 2903 [74] and C-H bending of the –CH2– functional group at 1467cm^−1^ [75]. The peak of the symmetric methyl group is presented at 2831 cm^−1^ [75], while the peak attributed to -CH- rocking is at 711 cm^−1^ [74]. 

The FB, as a copolymer of polyethylene and maleic anhydride, shows two peaks in the FI-IR spectrum attributed to cyclic anhydrides at 1705 and 1735 cm^−1^ [72]. 

The peak at 1740 cm^−1^ presented in wood spectra probably corresponds to the breaking of carbonyl bonds C=O [76]. The chemical reaction between wood and maleic anhydride in WPC results in the formation of ester structures, which could be observed at three peaks around 1743 cm^−1^, 1255 cm^−1^, and 1000 cm^−1^ [72,76,77,78]. The band at 1000 cm^−1^ could also be connected with the RCH=CH2 functional group with a response on C–CH2 out-of-plane bending [74].

### 3.2. The Results of Thermal Analysis 

**Figure 5:**
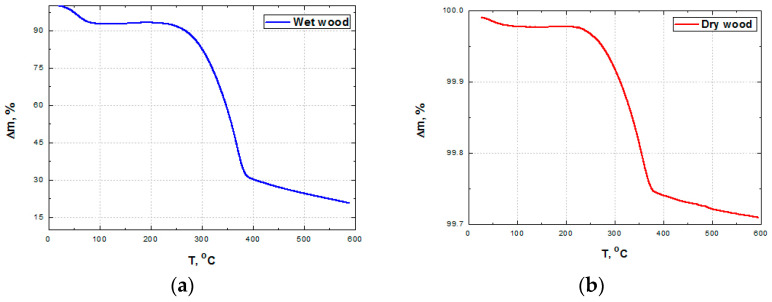
The weight % change with temperature for (**a**) wet wood and (**b**) dry wood.

The TG curves of dry wood and wet wood are presented in Figure 5 to present the hydrophilicity of wood flour. After the dehydration of wood flour in the temperature region between 45 °C and 100 °C, the process moves to dry matter decomposition in pyrolysis [79]. The high weight loss between 300 °C and 380 °C corresponds to the thermal decomposition of cellulose and hemi cellulose, as reported elsewhere [80,81].

**Figure 6:**
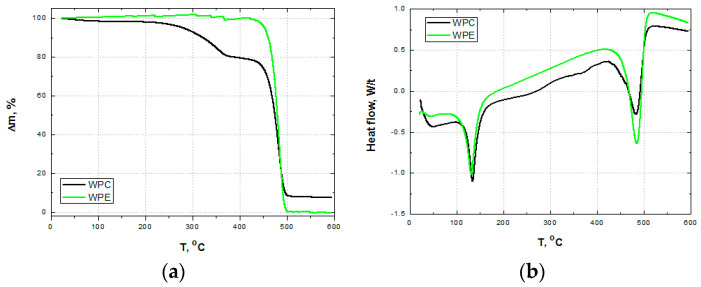
(**a**) TG and (**b**) DSC curves of wood–plastic composite and waste polyethylene.

The results of TG and DSC analysis for pure WPE and WPC (WPC60-0) are presented in Figure 6.

The first thermal decomposition temperature is around 131 °C, with a change in enthalpy values of 124.5 J/g, which corresponds to the melting point of WPE. The thermal degradation of both WPE and WPC starts at the temperature of 442 °C, increases to 502 °C, and reaches its maximum at 484 °C. The temperature peak of the change in enthalpy for degradation temperatures is 285.2 J/g [82].

### 3.3. Water Swelling and Water Absorption Analysis

**Figure 7:**
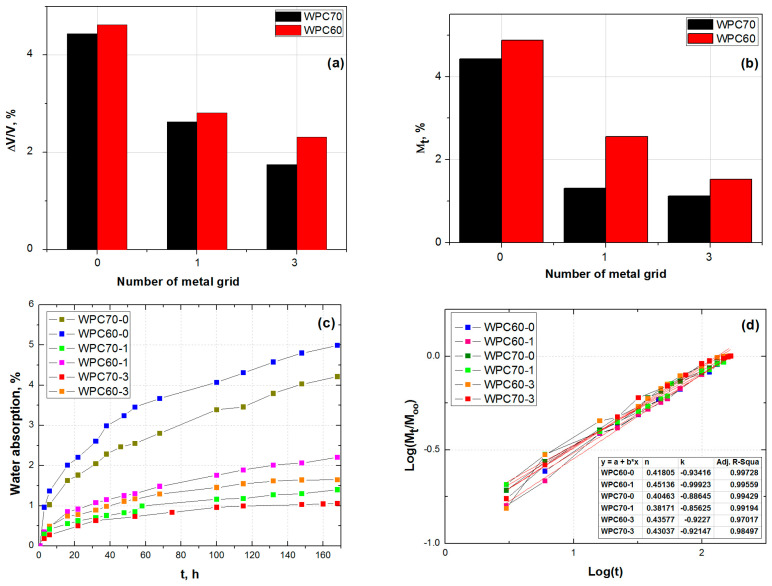
Results of (**a**) volume change, (**b**) weight change, (**c**) water absorption, and (**d**) diffusion parameters of WPC samples.

The absorption of water is monitored for 7 days, and the results of resistance to moisture absorption are presented in Figure 7. Figure 7a shows the volume change (swelling) caused by water absorption in the sample series of WPC60 and WPC70. Figure 7b shows the weight change of the sample series of WPC60 and WPC70. It is obvious from the presented histograms that the samples with a higher content of wood have higher swelling and higher water absorption due to the wood’s hydrophilic nature, as reported elsewhere [83,84]. In the case of pure WPC samples, the serial WPC60 has higher values of volume and mass change. Also, pure WPC samples (WPC60-0 and WPC70-0) have shown higher swelling and water absorption compared with WPC with one and three metal grids. A detailed analysis is presented in Figure 7c. The characteristic shape of the curves indicates that higher and faster absorption of the water is achieved in the first 12 h. After 30 h, the values have a slight increase, which means that the material has a tendency to become saturated.

According to [55], three different mechanisms could be responsible for the conduction of water into composite material. The type of water diffusion mechanism can be determined by analyzing the shape of the curves in Figure 7c or, more precisely, by applying Equation (4) [83,84]:

(4) $$\frac{M_{t}}{M_{\infty }}=k\cdot t^{n}$$

where *M_t_* is the content of the moisture at time *t*; *M_∞_* is the content of the moisture at equilibrium; *k* is a constant that describes the geometry of reinforcements and the material’s affinity to water molecules; and *n* is a constant that indicates one of the three types of transport mechanism. After taking the logarithm, Equation (4) becomes

(5) $$\log\left(\frac{{\bar{M}}_{t}}{{\bar{M}}_{\infty }}\right)=\log\left(k\right)+n\cdot \log\left(t\right)$$

In Equation (5), *n* represents the slope of the curves, while the parameter log(*k*) is the y-intercept in Figure 7d. The values close to 0.5 indicate Fick’s low diffusion mechanism. The results are presented in Figure 7d as the log–log dependence of water absorption and time of immersion. The values of n presented in Figure 7d are in the range between 0.38 and 0.45, similar to [55]. The results suggest that water transport in studied WPC composites is controlled by Fick’s law, consisting of a diffusion of water molecules within the wood fibers between WPE polymer chains [84].

The other two mechanisms—(I) the capillary flow of water molecules at the surface between the polymer matrix and wood fibers, and (II) the transport of water molecules by microcracks in the polymer—are explained in detail in [83].

According to the results of statistical analysis obtained by the analysis of variance (ANOVA) one-way test and Bonferroni test, the samples exhibited significant differences in the obtained water absorption values. In particular, there is a significant difference in water absorption between no metal grid composites WPC60-0 and WPC60-1, WPC60-3, WPC70-1, and WPC70-3 (all investigated samples reinforced with metal grids), as well as between no metal grid composite WPC70-0 and WPC60-1, WPC60-3, WPC70-1, and WPC70-3 (all investigated samples reinforced with metal grids), indicating the influence of composite formulations on water absorption capacity.

### 3.4. The Microstructure Analysis

**Figure 8:**
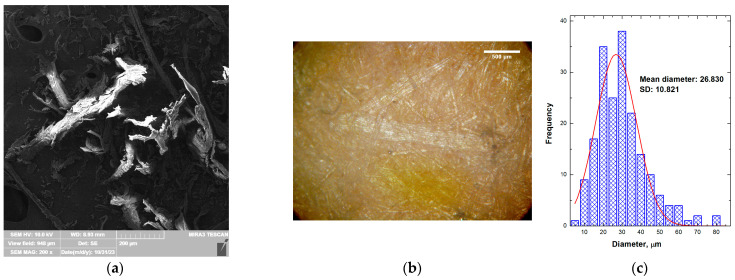
(**a**) FESEM, (**b**) micrograph, and (**c**) particle size histogram of wood flour.

The FESEM image and micrograph of wood flour used as WPC reinforcement are presented in Figure 8a and Figure 8b, respectively. The wood flour is composed of fibers, grains, and irregular-shaped particles. The particle distribution is presented in Figure 8c. The calculated mean diameter of grains and fibers is 26.810 μm (SD = 10.821). 

**Figure 9:**
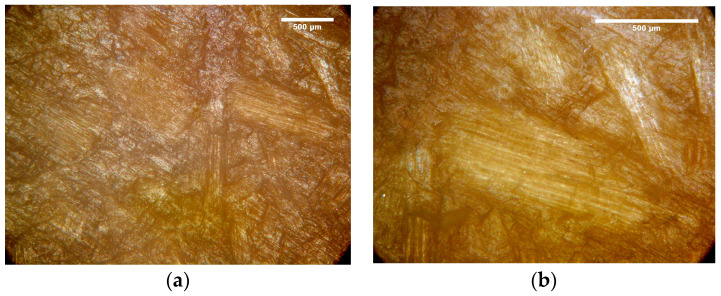
Micrographs of WPC60-0 with magnitude of (**a**) 50× and (**b**) 100×.

Micrographs of WPC60-0 with magnitudes of 50× and 100× are presented in Figure 9a and Figure 9b, respectively. The observations of the WPC surface indicate the existence of wood particles and a lot of fibers in the polymer matrix.

**Figure 10:**
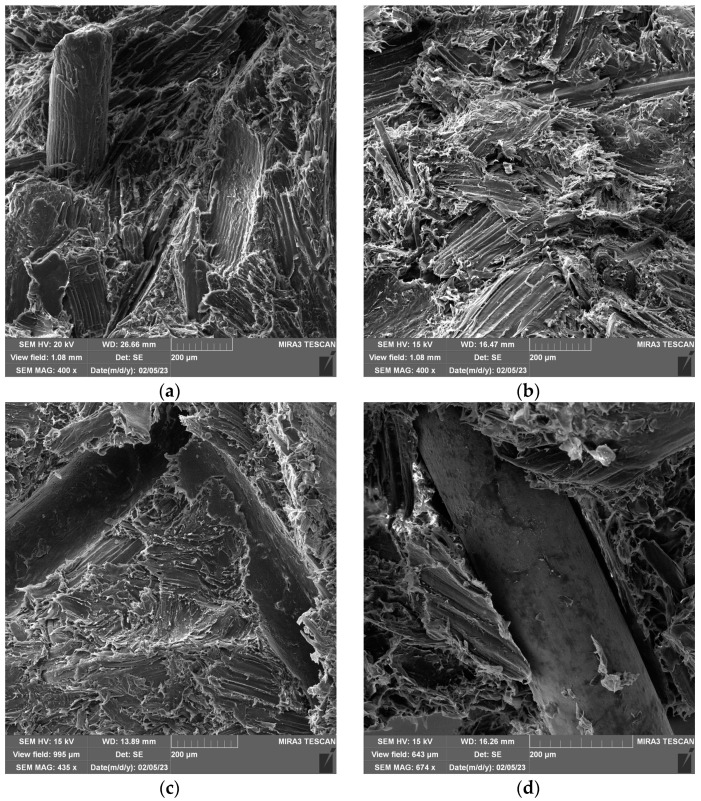
FESEM of samples: (**a**) WPC60-0, (**b**) WPC60-0a, (**c**) WPC70-3, and (**d**) WPC70-3a.

The microstructure of the fracture surfaces after HIT of specimens was examined using FESEM. Figure 10 shows FESEM images of fracture surfaces of the pure (without metal grid layers) WPC panel and WPC with three metal grid layers before and after immersion in water. Table 1 shows that the ratios of wood flour to polymer matrix are 30:70 and 40:60, but the mass of the sample is the same; this means a lower content of wood and polymer is applied to samples with metal grid layers. Consequently, by increasing metal grid layers, the absorption of water decreases. Also, the polymer phase covers the wood particles and fibers, which are responsible for swelling, and contributes to the water resistance of the composite [84]. On the other hand, the strength of a composite is dictated mostly by the quality of the bond at the interface, which allows stress transfer from the matrix to the filler. The fracture exists at the weakest bonding connection between the polymer matrix and the surface of the large wood fibers and grains. Small particles of wood flour are isolated by the polymer matrix and contribute to WPC stiffness, similar to [65].

FESEM analysis revealed apparent borders between wood and polymer, considering the orientation of wood fibers. It can be seen that the fracture was propagated like cleavage along the borders of pellets (Figure 10a,c). Immersion in water influenced the texture of wood fibers and the polymer–woodbond. FESEM images of immersed samples have pointed out that bonding in pellets became weaker and the cleavage propagated through them (Figure 10b,d). This is a consequence of considerable particle swelling during saturation. Wood fibers and particles are unstressed in dry conditions but swell during water absorption. Water fills up the gap at the fiber border, which is allowed by the large deformation of the compliant matrix [84]. 

It seems the bonding between WPC and metal grid after exposure to water becomes weaker (Figure 10d). A border is apparent between the polymer and the metal grid after water absorption (Figure 10d), while in Figure 10c before immersion, this border is not visible. It is a consequence of using only the pressure at the elevated temperature for bonding. The bonding strength properties could be improved using coupling agents, reinforced nano-particles, or appropriate additives.

### 3.5. The Impact Test

**Figure 11:**
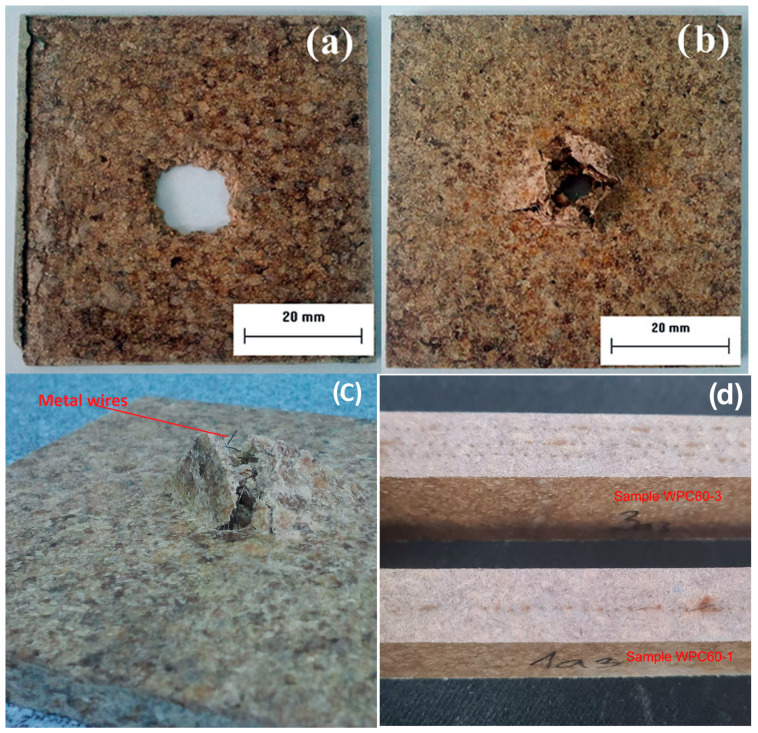
Samples of (**a**) WPC70-0 after impact test, (**b**) WPC70-3 after impact test, (**c**) WPC60-3 after impact test, and (**d**) the cross-section of WPC60-1 and WPC60-3.

The impact puncture test was performed on the samples with the aim of investigating the influence of composite structure and moisture absorption on the impact properties. The test was performed on the samples before and after water immersion. Samples after the impact test, presented in Figure 11, show a higher resistance of the material to impact for the sample reinforced with three grids (Figure 11b,c). The metal wires from the punctured reinforcement grid are visible in Figure 11c. The cross-section of hybrid WPC samples clearly shows one and three parallel metal grids in the composite matrix, which are responsible for the improved mechanical properties (Figure 11d).

**Figure 12:**
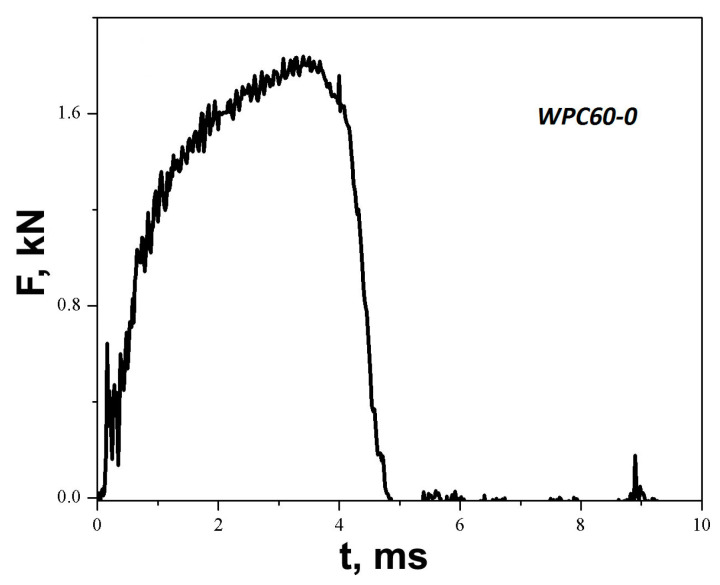
Force history of the impact test for WPC60-0.

The energy data were recorded for maximum load and total puncture. The characteristic force–time diagram for the sample of WPC60-0 is presented in Figure 12.

Similar diagrams are obtained directly from the test device for all samples, and the values of crack initiation energy, total energy, and ductility index before and after water immersion (assigned by subscript a) are presented in Table 2 and Table 3 for WPC60 and WPC70. The shape of the curve presented in Figure 12 indicates that the failure mode has changed from brittle/ductile to partially ductile [85]. The share of ductility increases for composites with metal layers (Table 2 and Table 3). The performance against impact perforation of the composites was also evaluated by the ductility index, *DI*:

(6) $$DI=\frac{E_{tot}-E_{init}}{E_{tot}}=\frac{E_{prop}}{E_{tot}}$$

where *E_prop_* = (*E_tot_* − *E_init_*) is the energy required to achieve full sample penetration after crack initiation, i.e., crack propagation, and demonstrate the post-maximum range [86,87].

It is obvious from Table 2 and Table 3 that the ductility index (*DI*) increases with the number of metal layers. The crack propagation strength *σ* increases with increased wood content and the number of metal layers. The load transfer in hybrid composites was improved by adding and multiplying the metal grids with WPC; thus, the composite strength was raised. Also, the higher content of wood fibers provides more fibers to facilitate load transfer. The dependence of the crack initiation energy *E_init_* on the wood content in hybrids was not significant because it is possible that it is a matrix-related parameter [87]. The total specific energy *E_total_* increased as the wood content and number of metal grids rose. All these impact parameters were decreased after water absorption.

**Figure 13:**
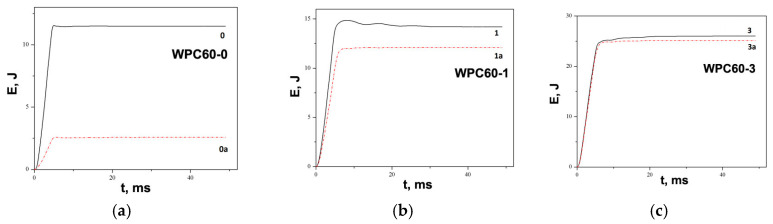
Energy absorption graphs of samples before and after water immersion: (**a**) WPC60-0, WPC60-0a, (**b**) WPC60-1, WPC60-1a, and (**c**) WPC60-3 and WPC60-3a.

**Figure 14:**
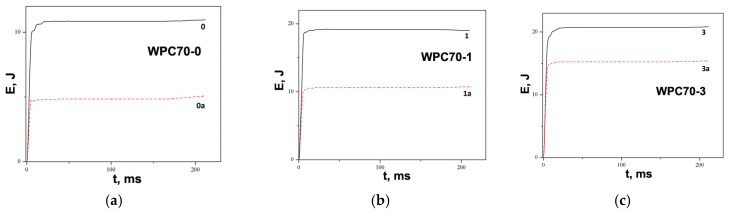
Energy absorption graphs of samples before and after water immersion: (**a**) WPC70-0, WPC70-0a, (**b**) WPC70-1, WPC70-1a, and (**c**) WPC70-3 and WPC70-3a.

Figure 13 and Figure 14 show energy–time curves for samples before and after water immersion. Energy was increased with a higher wood content in WPC. The impact test on WPC samples performed by Kofi et al. shows a maximum energy of 2.75 J and an absorbed energy of about 2 J for all tested samples, where the proportion of wood in polyethylene matrix varied from 10–40 wt. % [88]. In our work, the maximum energy has higher values, between 12 and 25 J, which depends on the composite structure. This difference in energies can be associated with the applied synthesis procedure, where, in addition to extrusion, hot pressing was used. Also, the big difference in energy values is due to different impact test methods. Furthermore, the absorbed energy is calculated and presented as the unit J/m due to the influence of the thickness of the sample, which is 7 mm. Most importantly, the incorporated metal grids significantly contribute to the impact resistance, as can be seen from the energy data in Table 2 and Table 3. Figure 11c clearly illustrates the damage of the sample with a metal grid, which has a higher resistance to the impact.

The addition of metal grids and raising the number of grids resulted in increased energy. After water absorption, the energy of samples decreased compared with that of samples without water immersion. Figure 13 and Figure 14 clearly show that the difference between the energies before and after water absorption decreased with an increasing number of metal grids. The volume content of the metal grid raised and the metal grids became the carriers of the loading.

### 3.6. The Three-Point Bending Tests

The results of three-point bending tests show significant changes in the mechanical properties of WPC samples with different grid contents. As shown in Table 4 and Table 5, the flexural strength “σ“ increases linearly with the addition of metal grids. Values in parentheses are standard deviations.

**Figure 15:**
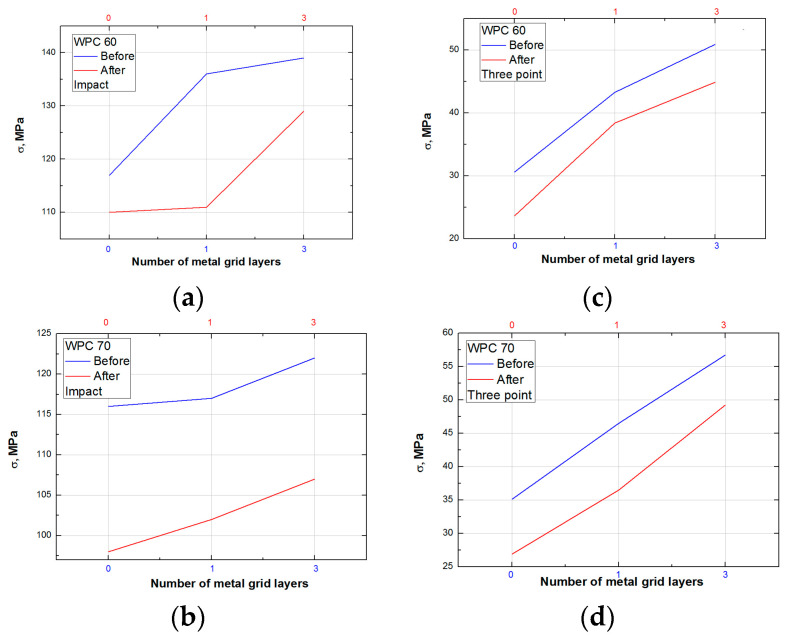
Comparison of mechanical properties. (**a**) HIT for WPC60; (**b**) HIT for WPC70; (**c**) maximum flexural strength for WPC60; (**d**) maximum flexural strength for WPC70.

**Figure 16:**
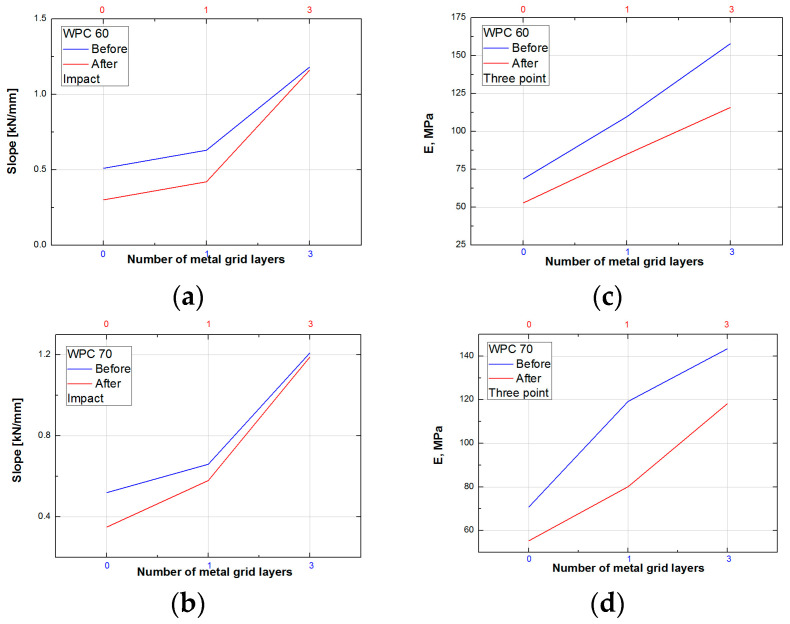
Comparison of modulus properties: (**a**) slope for WPC60; (**b**) slope for WPC70; (**c**) flexural modulus for WPC60; and (**d**) flexural modulus for WPC70.

Samples without metal grids “0” and “0a” have about 30% lower flexural strength than samples with one layer and 60% lower than samples with three metal grid layers. The influence of water swelling on the samples is about 20% for samples without a metal grid and decreases to 10% with the addition of metal layers (Figure 15 and Figure 16, Table 4 and Table 5). An increase in wood content in the samples slightly shows better flexural properties, consistent with the results of [8]. Moreover, the obtained values of maximum tensile strength for WPC60-0 and WPC70-0 are around 30 MPa, which are higher than those for WPC filled with 35 wt. %, and 45 wt. % of plastic (around 20 MPa), as reported elsewhere [8]. Similarly, an increasing trend of maximum tensile stress with wood filler content is reported elsewhere [89]. Also, according to [89], the values of max tensile strength are always slightly lower for recycled polyethylene. WPCs with a higher content of wood fillers show higher water absorption, which leads to higher differences between the tensile stress of samples before and after swelling. The addition of the Fusabond coupling agent and metal layers to WPC increased stress at failure and flexural strength compared to those of the WPC without reinforcement. The behavior of WPC samples after impact and three-point bending tests is summarized in Figure 15. Changes in the slope after the impact test and changes in the elasticity modulus after the three-point bending tests are shown in Figure 16.

The strength of a composite is dictated mainly by the bond quality between polymer and filler, allowing stress transfer from the matrix to the filler. The crack starts to grow at the weakest bonding points between the polymer matrix and the large wood fibers/particles [55]. Also, the region between the metal grid and the WPE matrix is weak, especially after the considerable particle swelling during saturation. Wood fiber/particles are unstressed in dry conditions but swell during water absorption. Water fills up the space at the fiber’s border, which is allowed by the large deformation of the compliant matrix. This phenomenon leads to the appearance of shear stresses at the interface between matrix and particles [84]. 

The modulus of elasticity increases with increasing wood content, consistent with the work of Koffi et al. (Tables 4 and 5) [88]. The lower values of the modulus of elasticity and tensile strength are evident for all samples after water immersion. The three-point tests show a little difference between the values before and after water treatment, in agreement with the results reported by [83]. Vice versa, the results of the impact test show that the differences in values for samples before and after water treatment are much greater. From this, it can be concluded that the samples treated with water show better resistance to exposure to flexural forces than to impact forces.

The results presented in Figure 16 show higher values for samples before water treatment, as expected. The modulus of elasticity increases almost two times with the addition of the three metal grids. Similarly, the results obtained by impact testing show that the slope increased more than two times for samples upgraded with three metal grids.

According to the obtained values of investigated parameters (Table 2, Table 3, Table 4 and Table 5), the ANOVA and Bonferroni test confirmed the influence of different WPC compositions on mechanical properties.

## 4. Conclusions

The results of this study show how the processing and use conditions affect the impact strength of hybrid WPC composite materials. The results of testing the samples on their resistance to moisture absorption indicate that a higher content of wood causes increased water absorption and swelling. SEM analysis revealed that water absorption influences the microstructure of composites due to their hydrophilic properties. An increased number of metal grid layers induces lower water absorption and swelling in both sample series, with 30 wt. % and 40 wt. % of wood content. The impact tests revealed that strength and total energy increased with wood content and a number of metal grids; the ductility index also increased in the same manner. The crack initiation energy has no dependence on the fiber content; it is a matrix-related parameter. After water absorption, the impact and flexural strength properties of the hybrid composite decreased. The higher (faster) energy decrease is characteristic of pure WPC. The metal grids as loading carriers led to a higher index of ductility, energy absorption, flexural module, and flexural strength. 

The results of the characterization of the synthesized hybrid WPCs show that the structural and mechanical properties as well as sustainability could be further improved by the incorporation of novel materials, such as recycled thermoplastics, natural reinforcements and fibers, nanocellulose, and biodegradable elements. Research activities could proceed to investigating the use of recycled composites and plastic matrix. The use of these materials will improve environmental protection and advance the development of green technology following the circular economy. Machining of WPCs, producing special geometries, finishing varnishes, impregnation layers, and refractory protection are specific topics that should receive more attention from researchers. Modern IT, like modeling, simulations, artificial intelligence, and machine learning, should be applied to predict and optimize the performance of manufactured WPCs [90].

## Figures and Tables

**Table 1:** The processing parameters of preparation of samples.

**SAMPLE**	**WPC60-0**	**WPC60-1**	**WPC60-3**	**WPC60-0a**	**WPC60-1a**	**WPC60-3a**
No of grid	0	1	3	0	1	3
m_grid_, (g)	0	10	30	0	10	30
x_WPE_, (%)	60	60	60	60	60	60
x_wood_, (%)	40	40	40	40	40	40
m_WPC_, (g)	400	390	370	400	390	370
x_WPC_ (%)	100	97.5	92.5	100	97.5	92.5
**SAMPLE**	**WPC70-0**	**WPC70-1**	**WPC70-3**	**WPC70-0a**	**WPC70-1a**	**WPC70-3a**
No of grid	0	1	3	0	1	3
m_grid_, (g)	0	10	30	0	10	30
x_WPE_, (%)	70	70	70	70	70	70
x_wood_, (%)	30	30	30	30	30	30
m_WPC_, (g)	400	390	370	400	390	370
x_WPC_ (%)	100	97.5	92.5	100	97.5	92.5

m_grid_ is the mass of incorporated grid; x_WPE_ is the mass percent of used WPE polymer matrix in WPC; x_wood_ is the mass percent of starting wood particles in WPC; m_wpc_ is the mass of wood, plastic, and additives (including Fusabond) without grid; and x_wpc_ is the mass percent of wood, plastic, and additives (including Fusabond) in produced WPC. It should be noted that the share of the cross-linking bonding agent Fusabond is 2 wt. % in all synthesized samples, while the content of other additives is neglected.

**Table 2:** Impact properties with standard deviations of WPC60 samples.

Sample	σ, (MPa)		*E_init_*, (kJ/m)		*E_tot_*, (kJ/m)		*DI*	Slope, (KN/mm)	
WPC60-0	117	(1.901)	1.29	(0.026)	1.76	(0.008)	0.27	0.51	(0.022)
WPC60-1	136	(2.070)	1.57	(0.013)	2.32	(0.022)	0.31	0.63	(0.026)
WPC60-3	139	(1.636)	2.06	(0.024)	3.12	(0.109)	0.49	1.18	(0.015)
WPC60-0a	110	(1.108)	0.67	(0.030)	0.88	(0.019)	0.24	0.30	(0.021)
WPC60-1a	111	(1.302)	1.67	(0.034)	2.10	(0.148)	0.20	0.42	(0.027)
WPC60-3a	129	(1.347)	1.43	(0.009)	3.10	(0.152)	0.54	1.16	(0.017)

**Table 3:** Impact properties with standard deviations of WPC70 samples.

Sample	σ, (MPa)		*E_init_*, (kJ/m)		*E_tot_*, (kJ/m)		*DI*	Slope, (KN/mm)	
WPC70-0	116	(1.246)	1.23	(0.019)	1.82	(0.008)	0.36	0.52	(0.021)
WPC70-1	117	(0.901)	1.15	(0.008)	1.99	(0.019)	0.37	0.66	(0.019)
WPC70-3	122	(1.961)	0.93	(0.016)	2.68	(0.021)	0.65	1.21	(0.013)
WPC70-0a	98	(0.830)	1.17	(0.015)	1.72	(0.009)	0.46	0.35	(0.028)
WPC70-1a	102	(0.412)	0.93	(0.019)	1.86	(0.011)	0.41	0.58	(0.031)
WPC70-3a	107	(0.716)	0.92	(0.023)	1.99	(0.011)	0.54	1.19	(0.023)

**Table 4:** The results of the three-point bending test for WPC60 composites.

Sample	F, (N)		σ, (MPa)		E, (MPa)	
WPC60-0	268.7	(0.564)	30.6	(0.329)	68.6	(0.502)
WPC60-1	372.1	(0.688)	43.3	(0.410)	109.7	(0.422)
WPC60-3	446.2	(0.450)	50.9	(0.526)	157.8	(0.370)
WPC60-0a	218.4	(0.339)	23.6	(0.492)	52.8	(0.541)
WPC60-1a	329.3	(0.358)	38.4	(0.305)	84.9	(0.311)
WPC60-3a	390.3	(0.268)	44.9	(0.259)	115.7	(0.274)

**Table 5:** The results of the three-point bending test for WPC70 composites.

Sample	F, (N)		σ		E, (MPa)	
WPC70-0	275.4	(0.406)	35.1	(0.313)	70.8	(0.502)
WPC70-1	379.2	(0.568)	46.5	(0.557)	119.2	(0.482)
WPC70-3	454.2	(0.316)	56.7	(0.432)	143.4	(0.286)
WPC70-0a	222.1	(0.661)	26.9	(0.297)	55.2	(0.453)
WPC70-1a	322.7	(0.466)	36.5	(0.240)	80.1	(0.327)
WPC70-3a	399.6	(0.469)	49.2	(0.472)	118.2	(0.291)

## Data Availability

Previously reported TG and FT-IR data, as well as Impact measurements data for some components of WPCs were used to support this study and are available at DOI: https://doi.org/10.1080/10426910903032212 and DOI: https://doi.org/10.2298/SOS1901115P. These prior studies (and datasets) are cited at relevant places within the text as references [37,69].
